# Cross species metabolic complementation of vitamin K2 biosynthesis determines emergent suppression of tomato *Fusarium* wilt

**DOI:** 10.1002/imt2.70186

**Published:** 2026-10-20

**Authors:** Lizhi Zhao, Yuhui Li, Jianning Zhao, Alexandre Jousset, Francisco Dini‐Andreote, Lu Tan, Xiujing Guan, Yan Xu, Zhong Wei

**Affiliations:** ^1^ Agro‐Environmental Protection Institute Ministry of Agriculture and Rural Affairs (MARA) Tianjin China; ^2^ Key Laboratory of Original Agro‐Environmental Pollution Prevention and Control, MARA Tianjin China; ^3^ Tianjin Key Laboratory of Agro‐Environment and Agro‐Product Safety Agro‐Environmental Protection Institute, MARA Tianjin China; ^4^ Jiangsu Key Laboratory of Organic‐based Fertilizer Creation and Soil Health Manipulation, Key Lab of Organic‐Based Fertilizers of China Nanjing Agricultural University Nanjing China; ^5^ Jiangsu Collaborative Innovation Center for Solid Organic Wastes, Educational Ministry Engineering Center of Resource‐saving fertilizers Nanjing Agricultural University Nanjing China; ^6^ Department of Plant Science & Huck Institutes of the Life Sciences The Pennsylvania State University University Park Pennsylvania USA; ^7^ The One Health Microbiome Center, Huck Institutes of the Life Sciences The Pennsylvania State University University Park Pennsylvania USA; ^8^ College of Agro‐Grassland Science Nanjing Agricultural University Nanjing China

## Abstract

Multi‐omics approaches have revealed a cross‐species functional emergence phenomenon leading to emergent suppression of *Fusarium* wilt. We show that a three‑species consortium exhibits emergent suppression of *Fusarium oxysporum* f. sp*. lycopersici* (Fol) that is absent in single or pairwise cultures. This antifungal activity arises from coordinated activation of two phylogenetically distinct vitamin K2 biosynthesis pathways—the *Men* pathway (primarily *Bacillus*) and the *Mqn* pathway (*Paenibacillus*)—whose complementary actions enhance K2 production and directly inhibit the pathogen. A structural analogue, vitamin K3, recapitulates this effect, offering translational potential. Our findings establish that emergent microbiome functions depend on interaction‑triggered latent metabolic activation, not constitutive exchange or additive effects.

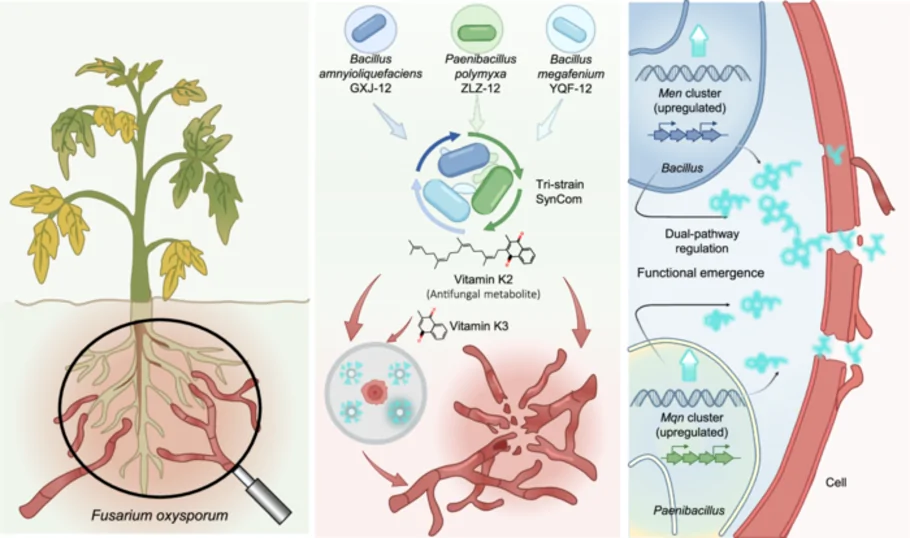

To the Editor,

Community‐level functions in microbial systems often arise from interspecies interactions rather than from the intrinsic properties of individual members, and therefore cannot be predicted from the performance of any single constituent [1, 2, 3]. Such interactions can give rise to emergent properties, whereby communities exhibit novel functions or enhanced collective performance [4]. Synthetic microbial community (SynCom) has emerged as a powerful experimental system to study such emergence, as coordinated interactions among defined species can generate functions that cannot be achieved by any single member in isolation [3, 5]. Although emergent functions or predictable emergent properties have been observed in a variety of SynCom configurations and in highly diverse constructed microbial communities [6], the mechanistic principles that govern their formation remain poorly understood. Current models often invoke mechanisms such as cross‐feeding or metabolic complementarity [7, 8], yet it remains unclear whether emergent functions arise from constitutive metabolite exchange among community members or from community‐dependent activation of metabolic pathways that are otherwise latent in individual taxa. Resolving this distinction is essential for understanding how community composition regulates metabolic function and gives rise to emergent phenotypes.

Soil‐borne diseases, such as those caused by *Fusarium oxysporum*, are suppressed through collective functions of rhizosphere microbial communities rather than by individual taxa [9, 10, 11]. Natural disease‐suppressive soils exemplify how complex consortia can collectively resist pathogen invasion, yet the metabolic mechanisms enabling such community‐level protection remain poorly understood. Single‐strain biocontrol agents often exhibit limited functional stability, whereas multispecies consortia display disproportionately strong and consistent effects [12, 13].

Here, we establish a defined rhizosphere SynCom (i.e., *Bacillus amyloliquefaciens* GXJ‐12, *Paenibacillus polymyxa* ZLZ‐12, and *Bacillus megaterium* YQF‐12) targeting Fol as a model system to dissect the metabolic regulation underlying functional emergence (Figure S1). By systematically comparing antifungal performance across single‐strain, pairwise, and full‐community systems, this work provides a mechanistic framework for understanding how multispecies interactions give rise to emergent functions in microbiomes.

## EMERGENT SUPPRESSION OF FOL IN A DEFINED TRI‐STRAIN SYNCOM

To investigate whether disease suppression can emerge as a community‐level property rather than an additive outcome of individual activities, we constructed SynComs using beneficial rhizosphere bacteria associated with Fol‐infected tomato soils. Metagenomic sequencing and Bray‐Curtis distance‐based PCoA analysis revealed a distinct shift in the microbial community structure of Fol‐infected tomato rhizosphere soil (PERMANOVA: *R* ^2^ = 0.425, *p* = 0.002; Figure S2A). Notably, in Fol‐infected tomato soils, several genera (e.g., *Paenibacillus*, *Bacillus*, *Streptomyces*, and *Lysobacter*) were identified as potential keystone taxa, as their abundances exhibited strong negative correlations with that of *Fusarium*, suggesting their potential roles in suppressing Fusarium wilt (Figure S2B). From these taxa, three culturable strains that exhibited strong in vitro antagonism against Fol were isolated, including *Bacillus amyloliquefaciens* GXJ‐12 (strain A), *Paenibacillus polymyxa* ZLZ‐12 (strain B), and *Bacillus megaterium* YQF‐12 (strain C) (Figure 1A,B).

**FIGURE 1:**
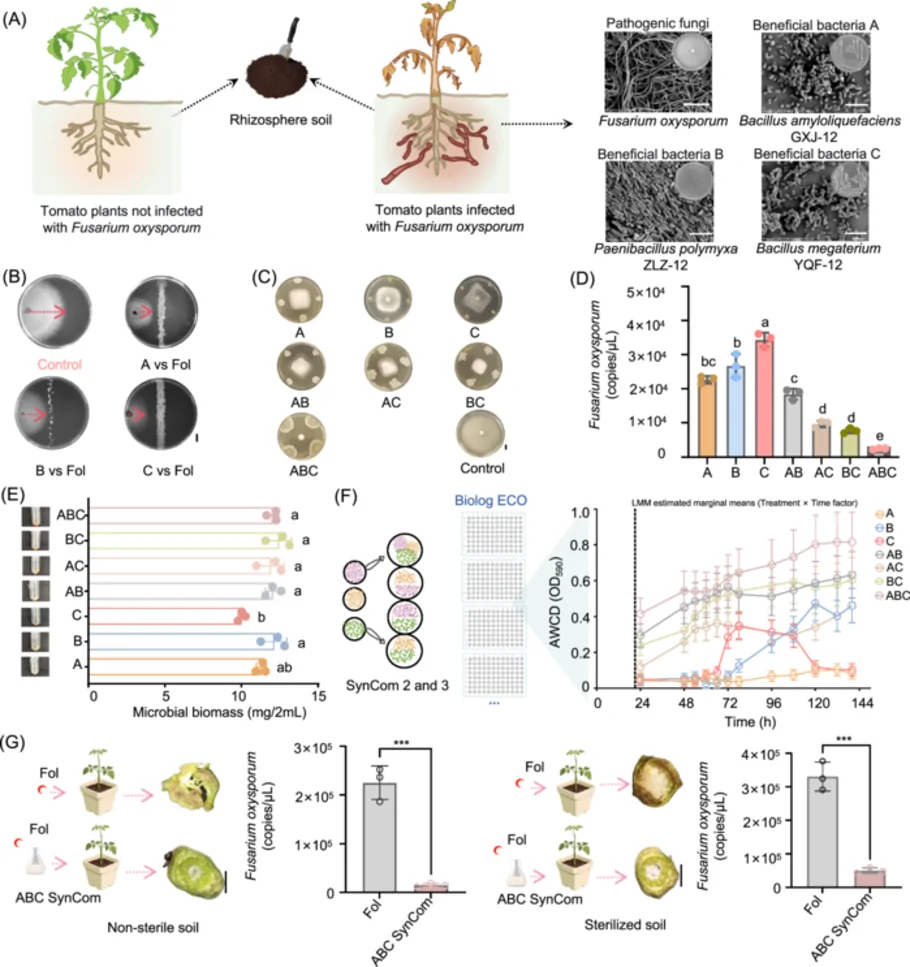
Biological and functional characterization of strains used to construct SynComs, and the emergent suppression of Fol by the ABC SynCom. (A) Morphological and scanning electron microscopy images of various microbial strains isolated from the rhizosphere soil of tomato plants infected with *Fusarium oxysporum*. *Bacillus amyloliquefaciens* GXJ‐12, *Paenibacillus polymyxa* ZLZ‐12, and *Bacillus megaterium* YQF‐12 are denoted as A, B, and C, respectively. Scale bar (*Fusarium oxysporum*) = 500 μm, Scale bar (strains A, B, and C) = 50 μm. (B) Antagonistic assays showing inhibitory effects of selected strains against Fol. Scale bar = 1 cm. (C) Representative images of Fol mycelial growth under single‐strain, pairwise, and tri‐strain SynCom treatments. Scale bar = 1 cm. (D) Quantification of Fol abundance by species‐specific qPCR (one‐way ANOVA with Tukey's HSD test, *p* < 0.05, *n* = 3). (E) Microbial biomass and resource utilization across treatments (one‐way ANOVA with Tukey's HSD test, *p* < 0.05, *n* = 3). (F) Time course of the Average Well Color Development (AWCD) of the microbial community under single‐strain and SynComs treatments (OD_590_). Symbols and error bars represent estimated marginal means (emmeans) ± 95% confidence intervals from a Linear Mixed effects Model (LMM), and lines connect the estimated marginal means across time points. Fixed effects included treatment, time, and treatment × time interaction (*n* = 3). (G) The control efficacy of the ABC SynCom against *Fusarium* wilt disease caused by Fol was demonstrated by quantifying Fol abundance via species‑specific qPCR and by examining sectioned tomato root and stem tissues for wilting symptoms, under both sterile and non‑sterile soil conditions (Welch's *t*‐test, *p < 0.05, **p < 0.01, and ***p < 0.001). Values with different lowercase letters are significantly different. AWCD: Average Well Color Development, a core indicator of overall microbial community metabolic activity in Biolog EcoPlate analysis. LMM: Linear Mixed effects Model, a regression model that combines fixed effects for population‐level relationships with random effects to account for variation and correlation within clustered or repeated‐measures data.

Using these strains, we assembled four SynComs comprising all pairwise combinations and a full tri‐strain consortium. Pairwise compatibility assays confirmed the absence of antagonistic interactions among the bacterial members, enabling stable community assembly (Figure S3). Importantly, the tri‐strain SynCom exhibited robust suppression of Fol, inhibiting mycelial growth by 94.74% and significantly outperforming all single‐strain and dual‐strain treatments (Tukey's HSD test, *p* < 0.05; Figures 1C and S4). This suppressive effect was accompanied by a marked reduction in Fol abundance, as quantified by species‐specific qPCR, with the strongest inhibition observed exclusively in the tri‐strain SynCom (Tukey's HSD test, *p* < 0.05; Figure 1D).

Notably, the enhanced antifungal activity of the tri‐strain SynCom could not be explained by simple increases in microbial growth. Compared with single strains and dual‐strain combinations, the ABC SynCom treatment did not show higher overall microbial biomass (Figure 1E). Nevertheless, the ABC SynCom exhibited an overall trend of superior resource utilization capacity, further suggesting that it may possess stronger metabolic capabilities [14] (Linear Mixed effects Model with Tukey adjustment, *p* < 0.05; Figures S5, S6, 1F and Table S1). These results demonstrate that Fol suppression is a strictly emergent function that arises only when all three bacterial members are present, rather than resulting from additive or density‐dependent effects.

## EMERGENT ANTIFUNGAL ACTIVITY IS MAINTAINED UNDER SOIL AND PLANT‐ASSOCIATED CONDITIONS

To evaluate whether the emergent suppressive function observed in vitro extends to more ecologically relevant contexts, we assessed SynCom performance in a soil suspension assay and in tomato pot experiments. Consistent with plate‐based assays, all SynComs inhibited Fol mycelial expansion relative to controls; however, the tri‐strain SynCom again exhibited the strongest effect, reducing mycelial area by 93.18% (Tukey's HSD test, *p* < 0.05; Figure S7).

In planta assays further demonstrated the functional relevance of this emergent property. Inoculation with the tri‐strain SynCom significantly alleviated *Fusarium* wilt symptoms and significantly reduced Fol abundance in tomato seedlings, irrespective of soil sterilization status (Welch's *t*‐test, *p* < 0.001; Figure 1G). We conducted preliminary field trials of the SynCom on tomato plants under natural *Fusarium* wilt pressure. The results showed that treatment with the ABC SynCom significantly reduced the incidence of tomato wilt disease by 21.00 ± 3.536% compared with the control group (Welch's *t*‐test, *p* < 0.01; Figure S8). Furthermore, field validation trials on strawberry also demonstrated that the triple ABC SynCom exhibited certain growth‐promoting effects (Figure S8), confirming that this synthetic microbial consortium has good broad‐spectrum crop adaptability and potential for field application. Together, these results confirm that the antifungal function of the tri‐strain SynCom is robust across environmental contexts and represents a biologically relevant emergent trait.

## VITAMIN K2 IS A KEY EFFECTOR METABOLITE UNDERLYING EMERGENT FOL SUPPRESSION

To identify the molecular basis of the emergent antifungal activity, we performed untargeted metabolomic profiling of culture supernatants from all treatments. Liquid Chromatography‐Mass Spectrometry analysis revealed extensive metabolic reprogramming in the tri‐strain SynCom relative to single‐strain and dual‐strain communities, with extracellular metabolites dominated by organic acids, heterocyclic compounds, and lipids (Figure S9). Differential abundance analysis identified 18 metabolites that were significantly enriched in the tri‐strain SynCom (Figure S10).

To prioritize metabolites strongly associated with antifungal efficacy, we applied random forest regression and correlation analyses. Among the top‐ranked features, several metabolites, including vitamin K2 (menaquinone), showed a significant positive association with Fol suppression (Figures 2A,B, S11 and Table S2). To functionally validate these predictions, four metabolites, vitamin K2, betaine, oleamide, and pantothenic acid, were selected for antifungal assays based on statistical importance, biosafety, and feasibility considerations (Figure 2C). All four compounds inhibited Fol growth, but only vitamin K2 exhibited the most pronounced effect, achieving an average inhibition rate of 87.42% (Tukey's HSD test, *p* < 0.05; Figure S12).

**FIGURE 2:**
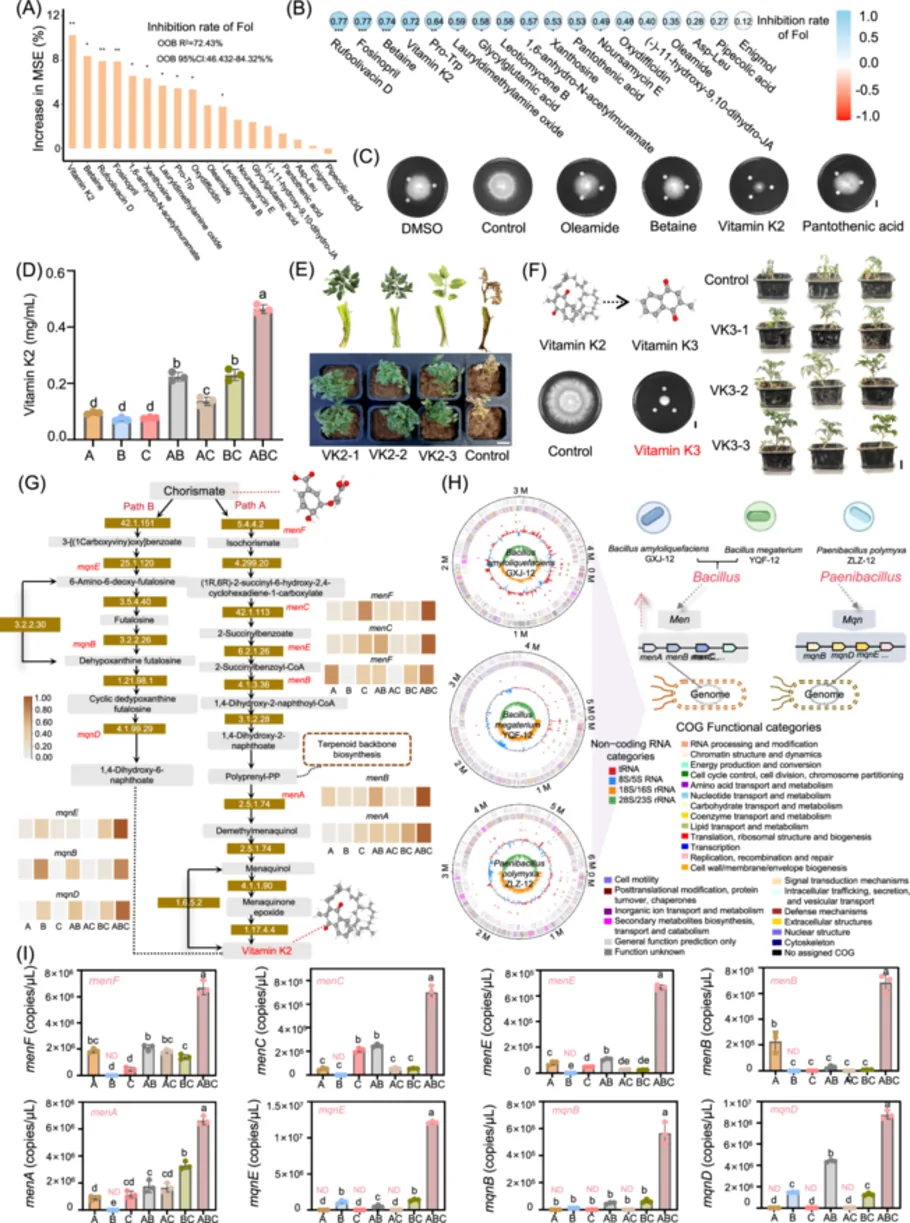
Identification of vitamin K as a key effector metabolite underlying emergent antifungal activity. (A) Variable importance of metabolites in predicting the inhibition rate of Fol based on random forest regression. The *y*‐axis represents the increase in mean squared error (%IncMSE), indicating the contribution of each metabolite to the model prediction. The 95% confidence interval was obtained via 2000 bootstrap resamples of the OOB predictions using the percentile method. Asterisks denote significant variables based on 1000 permutations (*p < 0.05, **p < 0.01, ***p < 0.001). (B) Correlation analysis between key metabolites and Fol inhibition rate (*n* = 21). Blue indicates positive correlation, red indicates negative correlation, and color intensity reflects correlation strength. Asterisks indicate significance (Spearman correlation; Benjamini–Hochberg FDR correction; *q < 0.05, **q < 0.01, ***q < 0.001). (C) Antifungal activity of selected metabolites against Fol. Scale bar = 1 cm. (D) Absolute quantification of vitamin K2 across treatments (one‐way ANOVA with Tukey's HSD test, *p* < 0.05). (E) Effect of exogenous vitamin K2 application on *Fusarium* wilt symptoms in tomato plants and histological analysis of vascular tissue integrity under different treatments. Vitamin K2 treatment concentrations were set at 0, 0.1, 0.2, and 0.4 mg/mL. The 0 mg/mL group was used as the control, and 0.1, 0.2, and 0.4 mg/mL were denoted as VK2‐1, VK2‐2, and VK2‐3, respectively. Scale bar = 5 cm. (F) Functional recapitulation of the emergent antifungal activity by vitamin K‐type quinones. Inhibition of Fol mycelial growth by vitamin K3 and disease symptoms in tomato plants treated with vitamin K3. Vitamin K3 treatment concentrations were set at 0, 0.1, 0.2, and 0.4 mg/mL. The 0 mg/mL group was used as the control, and 0.1, 0.2, and 0.4 mg/mL were denoted as VK3‐1, VK2‐2, and VK3‐3, respectively. Scale bars = 1 cm and 5 cm, respectively. (G) Distributed activation of complementary biosynthetic pathways enables elevated vitamin K2 production. Differential expression of genes associated with menaquinone biosynthesis (KEGG ko00130 pathway) across treatments. (H) Host origin of *Men* and *Mqn* pathway genes across SynCom members. Circular genome maps of three *Bacillus/Paenibacillus* strains, with concentric circles displaying genomic features from outermost to innermost: the outermost ring shows genome scale markers; the second and third rings display coding DNA sequences (CDSs) on the forward and reverse strands respectively, color‐coded by COG functional categories; the fourth ring indicates rRNA and tRNA locations; the fifth ring visualizes GC content, where outward red peaks represent regions with higher‐than‐average GC content (with peak height proportional to the deviation magnitude) and inward blue peaks denote regions with lower‐than‐average GC content; the innermost ring shows GC skew [(G − C)/(G + C)], where positive values indicate transcriptional preference on the forward strand while negative values suggest bias toward the rreverse strand. (I) cDNA absolute quantification PCR of *Men* (*menF, menC, menE, menB, menA*) and *Mqn* (*mqnE, mqnB, and mqnD*) gene expression across treatments. Data are presented as mean ± s.e.m (one‐way ANOVA with Tukey's HSD test, *p* < 0.05). Values with different lowercase letters are significantly different. CDSs: Coding DNA sequences, protein‐coding nucleotide sequences in the genome, typically starting with a start codon and ending with a stop codon. COG: Clusters of Orthologous Groups of proteins, a database of orthologous protein clusters constructed by comparing protein sequences from multiple complete genomes and grouping proteins from different species that share a common ancestor. GC: Guanine and Cytosine, the proportion of guanine and cytosine bases in genomic DNA, reflecting the base composition of the genome.

Absolute quantification further revealed that vitamin K2 production in the tri‐strain SynCom was 2.01–6.43‐fold higher than in single‐strain or dual‐strain treatments (Tukey's HSD test, *p* < 0.05; Figure 2D). Importantly, exogenous vitamin K2 addition significantly mitigated Fol‐induced tomato wilt in pot experiments. Histological analysis showed that vitamin K2 treatment preserved vascular bundle integrity and reduced tissue necrosis relative to Fol‐infected controls (Figure 2E). Together, these results identify vitamin K2 as a central effector linking community‐level metabolic output to emergent disease suppression.

## A LOW‐COST VITAMIN K‐TYPE QUINONE RECAPITULATES THE EMERGENT ANTIFUNGAL FUNCTION

To test whether the antifungal activity observed in the tri‐strain SynCom reflects a conserved functional property of vitamin K‐type quinones rather than a molecule‐specific effect, we applied vitamin K3, a structurally related analogue of vitamin K2. In vitro assays showed that vitamin K3 significantly inhibited Fol mycelial growth by more than 95%, recapitulating the antifungal activity of vitamin K2 (Figure 2F). It is noteworthy that the cost of vitamin K3 is significantly lower than that of vitamin K2, being merely 1/277th of its price (Figure S13), highlighting its potential utility as a scalable proxy for community‐derived metabolites.

To assess whether this functional equivalence extends to plant‐associated contexts, we performed pot experiments with exogenous vitamin K3 application. Consistent with the in vitro results, vitamin K3 treatment significantly alleviated *Fusarium* wilt symptoms and increased both aboveground and belowground tomato biomass compared to untreated controls (Tukey's HSD test, *p* < 0.05; Figures 2F and S14). These results demonstrate that antifungal activity is conserved across vitamin K‐type quinones, supporting a mechanism based on shared redox‐active physicochemical properties rather than compound‐specific biosynthesis. Previous studies have also found that these compounds participate in redox cycling and electron transport processes that disrupt fungal cellular homeostasis and oxidative balance [15, 16]. Mechanistic understanding of the quinone‐fungus interaction, particularly the in planta dynamics, remains the next critical step.

## DISTRIBUTED DUAL BIOSYNTHETIC PATHWAYS ENABLE HIGH‐LEVEL VITAMIN K2 PRODUCTION IN THE TRI‐STRAIN SYNCOM

We next investigated why vitamin K2 production was uniquely enhanced in the tri‐strain SynCom. Integrated metatranscriptomic and metabolomic analyses revealed significant enrichment of the KEGG ko00130 pathway, which governs ubiquinone and terpenoid quinone biosynthesis, in the tri‐strain treatment. Within this pathway, genes involved in menaquinone biosynthesis were strongly upregulated relative to all other treatments (Figure 2G).

Specifically, genes associated with the *Men* pathway (*menF, menC, menE, menB*, and *menA*) and the alternative *Mqn* pathway (*mqnE, mqnB*, and *mqnD*) both showed significantly higher expression in the tri‐strain SynCom (*p* < 0.05; Figure 2G). Additionally, genome‐wide functional annotation of these beneficial strains revealed enrichment of genes associated with cellular processes (e.g., cell wall and membrane biosynthesis and membrane transport), metabolic functions (e.g., carbohydrate and amino acid metabolism, as well as cofactor and vitamin metabolism), and molecular activities (e.g., catalytic activity, transporter activity, and binding) (Table S3 and Figures S15–S17). We constructed genome maps for each strain through whole‐genome sequencing. Host‐origin analysis revealed a clear partitioning, with the *Men* pathway genes primarily associated with *Bacillus* strains, whereas *Mqn* pathway genes originated from *Paenibacillus* members (Figure 2H). Quantitative PCR independently confirmed the upregulation of these genes in the tri‐strain SynCom relative to other SynComs (Tukey's HSD test, *p *< 0.05; Figures 2I, S18, S19 and Table S4).

Interspecies interactions such as cross‐feeding are commonly invoked to explain cooperative functions in microbial communities [17, 18]. These models typically assume constitutive metabolite production by individual members, with community‐level effects arising through metabolite exchange [19, 20]. Our findings instead support a mechanism in which community composition acts as a regulatory trigger for the coordinated activation of complementary biosynthetic pathways. These findings demonstrate that high‐level vitamin K2 production emerges from the coordinated activation of complementary biosynthetic pathways distributed across different species. Neither pathway alone, nor their partial activation in dual‐strain communities, was sufficient to generate comparable vitamin K2 levels, indicating that the tri‐strain SynCom enables an enhanced and integrated biosynthetic capacity through interspecies regulatory integration, collectively enhancing Fol suppression.

## CONCLUSION

This study demonstrates that a three‐species synthetic consortium suppresses fungal pathogen *Fusarium oxysporum* f. sp. *lycopersici* through a strictly emergent mechanism: the coordinated activation of two phylogenetically distinct vitamin K2 biosynthetic pathways (*Men* and *Mqn*) distributed across *Bacillus* and *Paenibacillus* species. Neither single strains nor pairwise combinations suffice, indicating that community‐dependent activation of latent pathways—not constitutive metabolite exchange—drives the antifungal effect. These findings establish cross‐species metabolic complementation as a mechanism for functional emergence, with vitamin K2 as a keystone effector metabolite. Future work should determine whether these metabolites act directly on plant defense or indirectly via pathogen inhibition, using spatially resolved approaches such as split‐root systems and metabolite tracing.

## AUTHOR CONTRIBUTIONS

**Lizhi Zhao**: Methodology; data curation; writing—original draft; formal analysis. **Yuhui Li**: Methodology; writing—original draft; visualization. **Jianning Zhao**: Methodology; data curation; writing—review and editing. **Alexandre Jousset**: Writing—review and editing. **Francisco Dini‐Andreote**: Writing—review and editing; funding acquisition. **Lu Tan**: Methodology; data curation. **Xiujing Guan**: Methodology; data curation. **Yan Xu**: Conceptualization; funding acquisition; project administration; writing—review and editing. **Zhong Wei**: Conceptualization; writing—review and editing.

## CONFLICT OF INTEREST STATEMENT

The authors declare no conflicts of interest.

## ETHICS STATEMENT

No animals or humans were involved in this study.

## Supporting information


**Figure S1:** Overview of the experimental design.
**Figure S2:** Effects of Fol infection on microbial community structure and inter‐species interactions.
**Figure S3:** Validation and evaluation of interbacterial antagonism.
**Figure S4:** Evaluation of the inhibitory activity against Fol mycelial growth.
**Figure S5:** LMM spline‐smoothed curves of estimated marginal means for AWCD (OD_590_) over time under different treatments.
**Figure S6:** Diversity index of carbon source utilization evaluated by the Biolog EcoPlate.
**Figure S7:** Inhibitory effects of each treatment on Fol mycelium growth in the presence of microbial community from simulated soil suspension.
**Figure S8:** Field trial results of the ABC SynCom system on tomatoes and strawberries.
**Figure S9:** Determination of extracellular metabolites of bacteria in different treatment groups.
**Figure S10:** Comparison of the major significantly different metabolites among different treatment groups.
**Figure S11:** Five‐fold cross‐validation results based on the random forest model.
**Figure S12:** Antifungal activity of selected metabolites against Fol.
**Figure S13:** Cost comparison between vitamin K2 and vitamin K3.
**Figure S14:** Comparison of tomato plant biomass among different treatments.
**Figure S15:** Functional gene annotation of *Bacillus amyloliquefaciens* GXJ‐12.
**Figure S16:** Functional gene annotation of *Paenibacillus polymyxa* ZLZ‐12.
**Figure S17:** Functional gene annotation of *Bacillus megaterium* YQF‐12.
**Figure S18:** Standard curves for absolute quantification of genes.
**Figure S19:** Melting curves for absolute quantification of genes.

**Table S1:** Pairwise comparisons of AWCD (OD_590_) among treatments at each time point based on a repeated‐measures linear mixed‐effects model.
**Table S2:** Statistical results of the correlation between key metabolites and Fol inhibition rate.
**Table S3:** Genomic characteristics of the three beneficial strains.
**Table S4:** Specific primer information of *Men*/*Mqn* gene cluster title.

## Data Availability

The data that support the findings of this study are available from the corresponding author upon reasonable request. Sequencing data are deposited in NCBI: PRJNA1437754 (https://www.ncbi.nlm.nih.gov/bioproject/PRJNA1437754), PRJNA1437844 (https://www.ncbi.nlm.nih.gov/bioproject/PRJNA1437844), PRJNA1437859 (https://www.ncbi.nlm.nih.gov/bioproject/PRJNA1437859), and PRJNA1437861 (https://www.ncbi.nlm.nih.gov/bioproject/PRJNA1437861). The data and scripts used are saved in GitHub (https://github.com/LZ-XXY/iMeta-2026-923). Supplementary materials (methods, figures, tables, scripts, graphical abstract, slides, videos, Chinese translated version, and updated materials) may be found in the online DOI or iMeta Science http://www.imeta.science/.
