# Supplementary material for: Cross species metabolic complementation of vitamin K2 biosynthesis determines emergent suppression of tomato Fusarium wilt
Source: Imeta. 2026 Oct 20:e70186. Online ahead of print. doi: 10.1002/imt2.70186 (PMC13683815; doi:10.1002/imt2.70186)
Supplement: Supplementary file 1 [file IMT2-9999-e70186-s002.docx]

Supporting information to

**Cross species metabolic complementation of** **vitamin K2 biosynthesis determines emergent suppression of tomato *Fusarium* wilt**

**Running title:** Interspecies functional emergence inhibits wilt

Lizhi Zhao^1#^, Yuhui Li^1#^, Jianning Zhao^1, 2, 3^, Alexandre Jousset^4, 5^, Francisco Dini-Andreote^6, 7^, Lu Tan^1, 2, 3^, Xiujing Guan^1^, Yan Xu^1, 2, 3*^, Zhong Wei^4, 5, 8*^

^1^Agro-Environmental Protection Institute, Ministry of Agriculture and Rural Affairs (MARA), Tianjin 300191, China

^2^Key Laboratory of Original Agro-Environmental Pollution Prevention and Control, MARA, Tianjin 300191, China

^3^Tianjin Key Laboratory of Agro-Environment and Agro-Product Safety, Agro-Environmental Protection Institute, MARA, Tianjin 300191, China

^4^ Jiangsu Key Laboratory of Organic-based Fertilizer Creation and Soil Health Manipulation, Key Lab of Organic-Based Fertilizers of China, Nanjing Agricultural University, Nanjing 210095, China

^5^Jiangsu Collaborative Innovation Center for Solid Organic Wastes, Educational Ministry Engineering Center of Resource-saving fertilizers, Nanjing Agricultural University, Nanjing, 210095 China

^6^Department of Plant Science & Huck Institutes of the Life Sciences, The Pennsylvania State University, University Park, PA 16802, USA

^7^The One Health Microbiome Center, Huck Institutes of the Life Sciences, The Pennsylvania State University, University Park, PA 16802, USA

^8^College of Agro-Grassland Science, Nanjing Agricultural University, Nanjing 210095, China

^#^These authors contributed equally: Lizhi Zhao, Yuhui Li

*Correspondence: Yan Xu (xuyan@caas.cn); Zhong Wei (weizhong@njau.edu.cn).

**METHODS**

**Experimental design and sampling**

Tomato plants and associated rhizosphere soils were collected from a long-term greenhouse-based tomato-cucumber rotation experimental platform located in Wuqing District, Tianjin, China (117°2'46″E, 39°32'5″N). The site has been maintained under continuous vegetable rotation, providing a stable agroecosystem context for examining rhizosphere microbial conditions under pathogen pressure. Plants were classified as Fol-infected only if they exhibited whole-plant wilting symptoms and Fol was successfully re-isolated from stem tissues using standard pathogen isolation procedures. For each condition (infected and uninfected), six biologically independent plants were sampled as replicates. Individual plants, together with their tightly adhering rhizosphere soil (operationally defined as soil firmly attached to roots after gentle shaking), were processed independently. All samples were transported to the laboratory on dry ice within the same day of collection. Samples designated for DNA extraction were stored at −80 °C, whereas samples intended for microbial cultivation were stored at 4 °C and processed within 48 h of collection.

**Metagenomic sequencing**

Soil DNA extraction was performed using the MagBeads FastDNA Kit for Soil (MP Biomedicals, CA, USA) [1], following the manufacturer’s instructions, and stored at −20 °C prior to further analysis. DNA quantity and quality were assessed using a QubitTM 4 Fluorometer (Invitrogen, Carlsbad, CA, USA) and agarose gel electrophoresis, respectively. The extracted microbial DNA was processed to construct metagenome shotgun sequencing libraries with insert sizes of 400 bp by using Illumina TruSeq Nano DNA LT Library Preparation Kit. Each library was sequenced by Illumina NovaSeq X Plus platform (Illumina, San Diego, CA, USA) with PE150 strategy at Personal Biotechnology Co., Ltd (Personal Biotechnology Co., Ltd, Shanghai, China). Raw sequencing reads were processed to obtain quality-filtered reads for downstream analysis. Fastp (v0.23.2) [2] were used to remove the sequencing adapters, to trim low-quality reads. Assembly of reads for each sample was performed using MEGAHIT (v1.1.2) software. Using Prodigal (v2.6.3) [3] software to identify open reading frames (ORFs) and predict the coding regions to obtain corresponding gene and protein sequence files. At the same time, using the cluster module of MMseq (version13.45111) software to remove redundancy and obtain non redundant gene and protein sets.

For the obtained non redundant protein set, species annotation was performed using mmseq2 software. The database contains sequences of prokaryotic and eukaryotic microorganism from NCBI-nr. Use the Strobealign (version 0.12.0) plugin to map the high-quality reads of each sample onto the overlapping group, and make the featureCounts (version 2.0.3, http://subread. sourceforge.net/). Count reads to determine gene abundance.

**Isolation of culturable bacterial and fungal strains**

Rhizosphere samples used for strain isolation in this study were collected from a tomato cultivation base in Wuqing District, Tianjin, China (117°2'46″E, 39°32'5″N). Bacterial strains were isolated from the rhizosphere soil of tomato plants infected with the fungal pathogen Fol, whereas the pathogenic fungal strain (Fol) was isolated from the root and stem tissues of tomato plants exhibiting wilt symptoms. Bacterial isolation was performed using the dilution plating method [4]. Briefly, 10 g of rhizosphere soil was suspended in 90 mL of sterile deionized water and thoroughly homogenized. The suspension was serially diluted (10^-2^, 10^-3^, and 10^-4^), and 100 µL of each dilution was spread onto Nutrient Agar (NA) plates. Plates were incubated in an inverted position at 30 °C for 48 h. Three replicate plates were prepared for each dilution. Individual colonies were selected and purified through three successive streaking steps to obtain pure cultures. Purified bacterial strains were stored long-term at −80 °C in 30–40% glycerol stocks.

Fungal isolation of the fungal strain was performed using a plant tissue isolation method [5]. Diseased tissues were surface-sterilized by immersion in 75% ethanol for 20 s, followed by three rinses with sterile water, then immersed in a 5% sodium hypochlorite solution for 5 minutes and rinsed five times with sterile water. Sterility of the final rinse was confirmed by plating aliquots onto agar medium. Surface-sterilized tissue fragments were then placed onto Potato Dextrose Agar (PDA), with three tissue pieces per plate, and incubated in an inverted position at 30 °C for 7 days to allow colony development. Emerging colonies were purified by single-spore isolation. Purified fungal strains were stored long-term at −80 °C in 30–40% glycerol stocks.

All isolated strains were deposited in the China General Microbiological Culture Collection Center (Preservation number: CGMCC; accession numbers: CGMCC No. 33171, CGMCC No. 33227, CGMCC No. 33172, and CGMCC No. 41736).

**Preparation of biological samples for scanning electron microscopy (SEM)**

Several pieces of approximately 1 mm^3^ tissue were excised from fresh samples and immediately fixed in 2.5% glutaraldehyde and placed at 4 °C for 12–24 h. The fixative solution was then removed, and the samples were rinsed three times with phosphate-buffered saline (PBS; pH 7.0) for 15 min each. Samples were subsequently dehydrated through a graded ethanol series (gradient dehydration). The dehydrated samples were placed in a critical-point dryer for drying. Dried samples were mounted onto sample stubs using conductive carbon adhesive and coated with platinum (Pt) using an ion sputter coater for 120 s. Samples were examined using a scanning electron microscope (SEM; Hitachi SU8100), and images were acquired [6].

**Whole genome sequencing of isolated bacterial and fungal strains**

To improve the strain identification and characterize the biological properties of the isolated strains, whole genome sequencing was performed on purified cultures. Genomic DNA from bacterial and fungal strains was extracted using the PureLink^®^ Genomic DNA Kit (Invitrogen, Carlsbad, CA, USA) and the Fungal DNA Kit (Omega Bio-Tek, Norcross, GA, USA), respectively. DNA quantity and quality were assessed using a NanoDrop ND-1000 spectrophotometer (Thermo Fisher Scientific, Waltham, MA, USA). Genome sequencing was performed using both PacBio and Illumina platforms. Raw sequencing reads were trimmed and quality-filtered using Trimmomatic (v0.36) to generate clean reads. Genome assembly and circularization were conducted using Unicycler and Circlator [7,8]. Functional annotation was performed using the KEGG and Gene Ontology (GO) databases.

**Antagonism assay to evaluate the suppressive effects of SynComs on Fol**

Antagonism assays were conducted to quantify the inhibitory effects of SynComs on Fol by measuring mycelial growth [9,10]. Reduced mycelial growth indicates stronger antagonistic activity of the bacterial strains or the synthetic communities. Under sterile conditions, individual strains or SynComs were first inoculated onto solid medium plates and incubated for 48 h. Subsequently, a 5 mm diameter agar plug of the pathogenic fungus was placed at the center of each plate. Plates were incubated at 30 °C for 7 days, after which the diameter of the Fol mycelial colony was measured. Mycelial area was quantified using ImageJ software [11,12].

**DNA extraction and absolute quantification PCR (Fol)**

Total microbial genomic DNA samples were extracted using the OMEGA Mag-Bind Soil DNA Kit (M5635-02) (Omega Bio-Tek, Norcross, GA, USA), following the manufacturer’s instructions, and stored at −80 °C prior to further assessment. Quantitative real-time PCR (qPCR) was per-formed using Fol specific primers (Primer-F 5'-TGGCTGTTCCTGAATTCTTATGC-3'; Primer-R 5'-TATTAACGAGAACGGCAGCG-3'). Using the AceQ reagent kit ® qPCR SYBR ® Green Master (Vazyme, Nanjing, China) conducts corresponding fluorescence quantitative PCR experiments. The reaction mixtures used for qPCR constitute of 8 uL of 2 × SYBR real-time PCR premixture, 0.5 uL (each) of primers F and R, and 8 uL of template DNA. qPCR amplifications were performed by using the following conditions: The PCR program consisted of an initial denaturation step of 5 min at 95 °C, followed by 40 cycles of denaturation at 95 °C for 10 s, annealing at 60 °C for 40 s followed. After the reaction is complete, analyze using LightCycler 480 II (Roche, Basel, Switzerland). PCR reaction fluorescence was plotted against the cycle number. The cycle where signal intensity crosses the threshold baseline is called the threshold cycle (C_t_). The C_t_ is used for estimating the initial DNA copy number.

**Determination of bacterial community metabolic activity**

To assess the carbon metabolic activity of single strains and other SynComs, this study employed the Biolog method. The Biolog ECO microplates (Biolog Inc., Hayward, CA, USA) used in the experiment contain 31 different carbon sources. Each well was inoculated with 150 μL of bacterial suspension (the initial optical density for all treatment groups was adjusted to OD_590_ = 0.1), followed by incubation in a light-proof incubator at 30 °C for 140 h. The average well color development (AWCD) at OD_590_ was measured using a microplate reader at different time intervals.

**Effect of ABC SynCom on tomato Fusarium wilt**

Sterilized and non-sterilized soils collected from the tomato cultivation base were used in this experiment. Plastic pots (upper diameter 37.2 cm, height 34.5 cm, bottom diameter 26.8 cm) were each filled with 25 kg of soil. Two treatments were established: (1) control, inoculated only with Fol; and (2) tri-strain SynCom treatment, inoculated with both the tri-strain SynCom and Fol. Each treatment included three biological replicates. The tri-strain SynCom was applied at a concentration of 1 × 10^6^ CFU g^-1^ soil. The Fol spore suspension was inoculated onto tomato roots at a concentration of 1 × 10^6^ CFU g^-1^ soil [13,14]. After 90 days, the susceptibility of tomato to Fol was assessed by qPCR quantification of Fol.

**Untargeted metabolomic analysis**

Extracellular metabolite was performed to investigate the metabolic mechanisms underlying SynCom-mediated inhibition of pathogenic fungi. Culture supernatants from SynCom-pathogen co-culture systems were collected by centrifugation at 8000 g for 10 min at 4 °C to remove bacterial and fungal cells. The resulting cell-free supernatants were stored at −80 °C prior to metabolomic analysis. Aliquots of the cell-free supernatant (100 μL) were transferred to 2 mL centrifuge tubes and mixed with 400 μL of ice-cold methanol-acetonitrile (1:1, v/v) for metabolite extraction. The mixture was vortexed for 30 s, incubated at −20 °C for 30 min, and centrifuged at 12,000 g at 4 °C for 10 min to precipitate proteins. The resulting organic extract (400 μL) was transferred to a new tube and dried completely under vacuum concentration. The residue was reconstituted in 150 μL of 50% methanol containing 5 μg/mL 2-chlorophenylalanine as an internal standard, vortexed for 30 s, and centrifuged again at 12,000 g at 4 °C for 10 min. The final supernatant was filtered through a 0.22 μm membrane into an LC vial for analysis. For quality control, equal volumes (10−20 μL) of each filtered sample were pooled to generate a QC sample.

Chromatographic separation was performed on an ACQUITY UPLC HSS T3 column (100 A, 1.8 μm, 2.1 mm × 100 mm) (Waters, Milford, MA, USA) maintained at 40 °C. The mobile phases consisted of 0.1% formic acid in water (A) andacetonitrile containing 0.1% formic acid (B). The elution gradient was as follows: 0−1 min, 5% B; 1−7 min, 5% to 95% B; 7−8 min, 95% B; 8−8.1 min, 95% to 5% B; 8.1−12 min, 5% B. The flow rate was 0.4 mL/min, and the injection volume was 2 μL.

Mass spectrometric data were acquired on a Thermo Orbitrap Exploris 120 mass spectrometer (Thermo Fisher Scientific, Waltham, MA, USA) equipped with a heated electrospray ionization (HESI) source, controlled by Xcalibur software (Thermo Fisher Scientific, Waltham, MA, USA). The instrument was operated in both positive and negative ion modes using data-dependent acquisition (DDA). The ESI source parameters were: spray voltage, +3.5 kV (positive) and −3.0 kV (negative); sheath gas flow rate, 40 arb; auxiliary gas flow rate, 15 arb; capillary temperature, 325 °C; auxiliary gas heater temperature, 300 °C. Full-scan MS data were acquired at a resolution of 60,000 over a mass range of m/z 100–1000. The top-4 most intense precursor ions were selected for higher-energy collisional dissociation (HCD) fragmentation at a normalized collision energy (NCE) of 30%. MS/MS spectra were acquired at a resolution of 15,000. Dynamic exclusion was set to 8 s. The automatic gain control (AGC) target and maximum injection time (IT) were set to Standard and 100 ms for full-scan MS, and to Standard and Auto for MS/MS, respectively.

Raw data were processed using Compound Discoverer 3.3 (Thermo Fisher Scientific, Waltham, MA, USA). Peak detection, alignment, and gap filling were performed using the default parameters of the software. Peaks with a detection rate below 50% across the QC samples were removed. Missing values for the remaining peaks were imputed using the Fill Gaps algorithm. Peak areas were normalized by the total summed peak area (sum normalization). Metabolite annotation was performed by matching accurate masses (MS1 tolerance set to 15 ppm) and MS/MS fragmentation spectra (match factor threshold set to 50) against the following spectral libraries: the in-house PSNGM Database, mzCloud (https://www.mzcloud.org/), LIPID MAPS (https://www.lipidmaps.org/), HMDB (https://hmdb.ca/), MoNA (https://mona.fiehnlab.ucdavis.edu/), and NIST 2020 MSMSy [15]. Metabolites were assigned confidence levels according to the Metabolomics Standards Initiative (MSI) guidelines. All qualitatively identified metabolites met level 2 or higher, based on accurate mass measurement (< 15 ppm error), tandem MS (MS/MS) spectral matching, and retention time consistency.

Pooled QC samples were analyzed at regular intervals (every 8–10 injections) throughout the LC-MS batch to monitor instrument stability. Metabolites with a relative standard deviation (RSD) ≥ 30% across QC samples were excluded from downstream analysis. In addition, principal component analysis (PCA) of all samples including QCs was performed to confirm clustering of QC samples and assess overall data reproducibility.

Vitamin K2 was identified as menaquinone-4 (MK-4, Menatetrenone; molecular formula C_31_H_40_O_2_) with a calculated monoisotopic mass of 444.30289 Da and observed [M + H]^+^ at m/z 445.31016 (mass error: 0.12 ppm), a retention time of 7.28 min, and MS/MS fragmentation matched to the mzVault spectral library (best match factor: 54.3; HMDB ID: HMDB0030017; KEGG ID: C00828). Additionally, based on the LC-MS analysis results, we conducted a comparative validation against reference standards and performed quantitative liquid chromatography analysis.

**Antagonistic experiment to test the suppressive effects of key metabolites on Fol**

The antifungal activity of four key metabolites (vitamin K2, oleamide, betaine, and palmitic acid) against Fol was evaluated using a disk diffusion assay. Briefly, sterile blank disks were soaked in the metabolite solutions (0.01 mg mL^-1^ in DMSO) for 30 min [16]. A 5 mm diameter agar plug of Fol was then placed at the center of a PDA plate, and the metabolite-soaked disks were positioned around the fungal inoculum. Plates were incubated at 30 °C for 5 days, after which the diameter of the Fol mycelial colony was measured. Mycelial area was quantified using ImageJ software [11,12], and inhibition rates were calculated to assess antifungal activity.

**Liquid chromatographic quantification of vitamin K2**

A vitamin K2 stock solution (100 mg L^-1^) was prepared, and a standard calibration curve was generated. For sample extraction, 1 mL of the sample was transferred into a 50 mL centrifuge tube, and 4 mL of extraction solvent (n-hexane: isopropanol, 2:1, v/v) was added and mixed thoroughly. The mixture was sonicated for 30 min, followed by centrifugation, and the supernatant was transferred into a 10 mL centrifuge tube. After evaporation under a nitrogen stream, the residue was re-dissolved in methanol and adjusted to a final volume of 1 mL. The solution was filtered through a 0.22 μm membrane filter prior to analysis. Sample analysis was performed using a Thermo Scientific U3000 HPLC system (Thermo Fisher Scientific, Waltham, MA, USA). Vitamin K2 concentrations were quantified using the standard calibration curve.

**Antagonistic experiment to test the suppression of vitamin K3 on Fol**

To test whether the antifungal activity observed in the tri-strain SynCom reflects a conserved functional property of vitamin K-type quinones rather than a molecule-specific effect, we applied vitamin K3, a structurally related analogue of vitamin K2. The antifungal activity of vitamin K3 against Fol was evaluated using a disk diffusion assay. Briefly, sterile blank disks were soaked in vitamin K3 solution (0.01 mg mL^-1^ in DMSO) for 30 min [16]. A 5 mm diameter agar plug of Fol was placed at the center of a PDA plate, and the soaked disks were positioned around the fungal inoculum. Plates were incubated at 30 °C for 7 days, after which the diameter of the Fol colony was measured. Mycelial area was quantified using ImageJ software [11,12], and inhibition rates were calculated to assess antifungal activity.

**Pot experiment to test the suppression of Fusarium wilt by vitamin K in tomato plants**

Sterile soil was used for the experiment. Plastic pots (top diameter 17.3 cm, height 12.5 cm, bottom diameter 13.5 cm) were filled with 4 kg of soil per pot. Based on vitamin K2 quantification results, treatment concentrations of vitamin K2 and vitamin K3 were set at 0, 0.1, 0.2, and 0.4 mg mL^-1^. Each treatment included three biological replicates. Vitamin K2 or vitamin K3 solutions were thoroughly mixed with the soil prior to transplanting tomato seedlings. Seedling roots were subsequently inoculated with a Fol spore suspension at a concentration of 1 × 10^6^ CFU g^-1^ soil. After 60 days, the growth status of tomato plants was recorded.

**Metatranscriptome analysis**

Total RNA was extracted from each sample using the RNA PowerSoil® Total RNA Isolation Kit (MoBio, Carlsbad, CA, USA) according to the manufacturer’s instructions [17]. The extracted RNA was assessed using 1.2% agarose gel electrophoresis and UV spectrophotometer for quality determination and quantification. Meanwhile, RNA integrity (RIN >= 6.5) was detected using an Agilent 2100 Bioanalyzer (Agilent, Santa Clara, CA, USA) [18]. After removal of ribosomal RNA using TIANSeq rRNA Depletion Kit (Tiangen, Beijing, China), Illumina’s TruSeq Stranded mRNA LT Sample Prep Kit (Illumina, San Diego, CA, USA) [19] was used for reverse transcription as well as metatranscriptome sequencing library construction. Each library was sequenced by Illumina NovaSeq X Plus (Illumina, San Diego, CA, USA) platform with PE150 strategy at Personal Biotechnology Co., Ltd (Personal Biotechnology Co., Ltd, Shanghai, China).

Raw sequencing reads were quality-filtered using fastp (v0.23.2). Then, ribosomal RNA was removed by BBTools-bbduk (v38.98) using its default rRNA reference database. Asembly of reads for each sample was performed using MEGAHIT (v1.1.2) software. Using Prodigal (v2.6.3) software to identify open reading frames (ORFs) and predict the coding regions to obtain corresponding gene and protein sequence files. Predicted coding sequences were translated into protein sequences and clustered to generate a non-redundant protein dataset using MMseqs2. Transcript abundance was normalized across samples, and functional annotation was performed by comparison against public databases.

**RNA extraction and cDNA absolute quantification PCR (*Men* and *Mqn* gene cluster)**

Total RNA samples were extracted with TriZOL reagent (Invitrogen, Carlsbad, CA, USA) [20]. The Advantage® RT-for-PCR Kit (Clontech, Mountain View, CA, USA) was used to generate the first strand cDNA according to the manufacturer’s instructions, and samples were stored at −80 °C prior to analysis. Gene-specific primer sets targeting *Men* and *Mqn* gene clusters were designed in this study (primer sequences are provided in Table S2). qPCR reactions were performed using the AceQ^®^ SYBR Green qPCR Master Mix (Vazyme, Nanjing, China). Each reaction contained 10 µL of 2 × SYBR Green real-time PCR premix, 0.5 µL of each primer, and 8 µL of template cDNA. Amplifications were carried out under the following conditions: For bacterial 16S rRNA – an initial denaturation step at 95 °C for 5 min, followed by 45 cycles of 95 °C for 15 s, and 60 °C for 30 s. Fluorescence signals were detected using a LightCycler 480 II (Roche Diagnostics, Basel, Switzerland). C_t_ values were determined based on fluorescence intensity crossing a defined baseline threshold and were used to estimate the initial gene copy numbers. Standard curves, amplification efficiencies, and melting curves for absolute quantification of *Men* and *Mqn* cluster genes are provided in the Supplementary Information (Figures S18 and S19).

**Antagonism experiment to test the synergistic effect of each SynCom**

To evaluate whether antagonism exists among the isolated Bacillus strains, which might affect the construction of synthetic bacterial communities, we further designed a bacterial antagonism test. Under sterile conditions, a sterilized toothpick was used to pick up a small amount of bacterial suspension and inoculate it onto the diagonal lines of a square solid agar NA (Nutrient Agar) medium. The inoculation points were gradually brought closer to form a “V” shape, with each treatment repeated three times. The antagonism between strains was assessed after incubating the plates upside down at 30 °C for 36–48 hours.

**Preparation of soil suspension**

Soil samples were collected from fertile, moist topsoil after removing the upper 3 cm surface layer. The collected soil was transferred into sterile beakers. Subsequently, a 10 g/L soil suspension was prepared by adding 1 g of soil to 99 mL of sterile distilled water in a 250 mL conical flask. The suspension was thoroughly mixed using a shaking incubator. To obtain a 10^-2^ dilution, a 0.5 mL aliquot of the resulting supernatant was transferred into a test tube containing 4.5 mL of sterile distilled water and subjected to serial dilution [21].

**Field verification of ABC SynCom**

To verify the application efficacy of the synthetic community under actual field production conditions, this study conducted field trials separately on two economic crops, tomato and strawberry. The experimental site was located in Wuqing District, Tianjin, China. The experiment included two treatments: Control (no ABC SynCom inoculation) and ABC SynCom (with ABC SynCom inoculation), with three replicates per treatment. After ABC SynCom powder was reconstituted and activated prior to application. It was administered as a root drench at a rate of 1 kg per mu at three critical growth stages, such as transplanting, flowering, and fruit enlargement. Plant growth parameters and disease incidence were measured at harvest.

**Determination of tomato biomass**

Aboveground and belowground biomass of tomato plants was determined using the oven-drying method. Whole plants were carefully removed from the growing medium, and roots were gently rinsed with clean water to remove adhering soil, followed by blotting with absorbent paper to remove surface moisture. Plants were separated into aboveground (stems and leaves) and belowground (roots) components at the stem base using scissors, and each fraction was placed in separate paper envelopes. Samples were initially heated at 105 °C for 30 min to halt metabolic activity, followed by drying at 80 °C to constant weight (Yamato Scientific Co., Ltd., Yamato, Japan) [22]. After drying, the samples were cooled to room temperature in a desiccator. Dry weights of aboveground and belowground biomass were measured using an analytical balance with 0.0001 g precision (Sartorius, Göttingen, Germany).

**Main Reagents**

Oleamide (CAS 301-02-0; powder; MACKLIN, Shanghai, China), Betaine (CAS 107-43-7; powder; MACKLIN, Shanghai, China), vitamin K2 (11032-49-8; powder; MACKLIN, Shanghai, China), Pantothenic Acid (CAS 79-83-4; powder; Aladdin, Shanghai, China), the solvent DMSO (CAS 200-664-3; powder; MACKLIN, Shanghai, China), vitamin K3 (CAS 58-27-5; powder; MACKLIN, Shanghai, China).

**Statistical analysis**

To investigate the effects of Fol infection on microbial community structure and inter-species interactions. Beta diversity was evaluated using the Bray-Curtis distance matrix and visualized via Principal Coordinate Analysis (PCoA), while differences in community structure between the Uninfected and Fol-infected groups were tested using Permutational Multivariate Analysis of Variance (PERMANOVA). Furthermore, Spearman's rank correlation analysis was performed on the top 50 most abundant genera within the Fol-infected group, and the results were visualized as a correlation heatmap. All statistical analyses and visualizations were conducted in R using the vegan, ggplot2, and corrplot packages, with a significance level of *p* < 0.05. AWCD data analysis was performed using R software. A repeated-measures linear mixed-effects model (LMM) was used. Treatment, Time, and the Treatment × Time interaction were included as fixed effects. ID, representing independent culture batches/microplates, was included as a random effect to account for the non-independence of repeated measurements within the same batch. Residual autocorrelation between adjacent time points within the same culture batch was modeled using a continuous first-order autoregressive (CAR1) covariance structure. Time was included in the model in two ways: 1) as a categorical variable for post hoc comparisons among treatments at each time point; and 2) as a continuous variable fitted with a restricted cubic spline (df = 3) to describe the overall nonlinear temporal trajectory. Fixed effects were estimated using REML and tested with Type III ANOVA. If the Treatment × Time interaction was significant, emmeans was used to calculate the estimated marginal means for each treatment at each time point, and pairwise comparisons were performed with Tukey adjustment; *p* < 0.05 was considered statistically significant. Mycelial area was quantified using ImageJ software, and inhibition rates were calculated to assess antifungal activity. The main bar charts and data analysis were completed using GraphPad Prism 10 and Origin 2024. Heatmaps showing variation in metabolite abundance across treatments were generated using TBtools. LEfSe analysis enables comparisons across multiple groups to identify metabolites with significantly different abundances among groups. LEfSe was chosen because it integrates non-parametric testing (Kruskal-Wallis and pairwise Wilcoxon) with effect size estimation (LDA score), making it suitable for metabolomics data that often exhibit non-normal distributions. This approach identifies metabolites that are not only statistically significant but also biologically consistent across multiple groups [23,24]. Random forest regression was performed in R software using the randomForest, rfPermute, boot, and ggplot2 packages. The random forest regression was performed on 21 treatments (7 treatments × 3 replicates), with the Fol inhibition rate as the response variable and metabolite abundances as predictors. Model performance was assessed by OOB R², RMSE, and MAE, with the 95% CI of OOB R² obtained from 2,000 bootstrap resamples. The model comprised 500 decision trees, and feature importance was quantified by permutation importance (% IncMSE) with significance assessed by 1,000 permutation tests. To evaluate the stability and feature importance of the random forest model, we further conducted five-fold cross-validation. According to the cross-validation curve, retaining four important variables is suggested to obtain ideal regression results. We validated and evaluated the degree of influence of the top four important variables on the Fol inhibition rate and their importance ranking; the ranking was derived from the random forest importance ranking based on the full dataset. For the correlation analysis between multiple metabolites and the inhibition rate, 18 metabolites were tested, with 21 measurements for each metabolite and 21 measurements for the Fol inhibition rate. The total number of tests was n-tests = 18. Multiple comparisons were corrected using the Benjamini-Hochberg false discovery rate (BH-FDR) method, and an adjusted *q* value < 0.05 was considered statistically significant. The correlations and their significance were visualized using a correlation heatmap. Genomic circular plots were generated using the circlize package in R. Statistical significance among groups was determined using one-way analysis of variance (ANOVA), the Tukey’s HSD post-hoc test, or the Welch’s *t*-test. A threshold of *p* < 0.05 was considered statistically significant.

**REFERENCES**

1.	Shi, Yang, Wenlan Lei, Yunmu Xiao, Tengfei Wu, Xuezhi Yang, Yong Li, Qian Liu, et al. 2026. “Photovoltaic coverage alters soil ecosystem multifunctionality: Contrasting effects and underlying mechanisms.” *Environmental Science and Technology* 60: 17158-17170. https://doi.org/10.1021/acs.est.5c14527

2.	Chen, Shifu, Yanqing Zhou, Yaru Chen, Jia Gu. 2018. “fastp: an ultra-fast all-in-one FASTQ preprocessor.” *Bioinformatics* 34: i884-i890. https://doi.org/10.1093/bioinformatics/bty560

3.	Hyatt, Doug, Gwo-Liang Chen, Philip F. LoCascio, Miriam L. Land, Frank W. Larimer, Loren J. Hauser. 2010. “Prodigal: prokaryotic gene recognition and translation initiation site identification.” *BMC Bioinformatics* 11: 119. https://doi.org/10.1186/1471-2105-11-119

4.	Thoenen, Lisa, Caitlin Giroud, Marco Kreuzer, Jan Waelchli, Valentin Gfeller, Gabriel Deslandes-Hérold, Pierre Mateo, et al. 2023. “Bacterial tolerance to host-exuded specialized metabolites structures the maize root microbiome.” *Proceedings of the National Academy of Sciences* 120: e2310134120. https://doi.org/10.1073/pnas.2310134120

5.	Bai, Yang, Daniel B. Müller, Girish Srinivas, Ruben Garrido-Oter, Eva Potthoff, Matthias Rott, Nina Dombrowski, et al. 2015. “Functional overlap of the *Arabidopsis* leaf and root microbiota.” *Nature* 528: 364-369. https://doi.org/10.1038/nature16192

6.	Ma, Zhiyuan, Meitong Jiang, Chaoyang Liu, Ertao Wang, Yang Bai, Mengting Maggie Yuan, Shengjing Shi, et al. 2024. “Quinolone-mediated metabolic cross-feeding develops aluminium tolerance in soil microbial consortia.” *Nature Communications* 15: 10148. https://doi.org/10.1038/s41467-024-54616-0

7.	M. Bolger, Anthony, Marc Lohse, Bjoern Usadel. 2014. “Trimmomatic: a flexible trimmer for Illumina sequence data.” *Bioinformatics* 30: 2114-2120. https://doi.org/10.1093/bioinformatics/btu170

8.	Xu, Mengyang, Lidong Guo, Shengqiang Gu, Ou Wang, Rui Zhang, Brock A Peters, Guangyi Fan, et al. 2020. “TGS-GapCloser: A fast and accurate gap closer for large genomes with low coverage of error-prone long reads.” *Gigascience* 9: giaa094. https://doi.org/10.1093/gigascience/giaa094

9.	Zhou, Xin, Jinting Wang, Fang Liu, Junmin Liang, Peng Zhao, Clement K. M. Tsui, Lei Cai. 2022. “Cross-kingdom synthetic microbiota supports tomato suppression of *Fusarium* wilt disease.” *Nature Communications* 13: 7890. https://doi.org/10.1038/s41467-022-35452-6

10.	Zhang, Shengquan, Peng-Lin Wei, Yuanyuan Li, Zedong Ren, Jie Fan, Wen-Bing Yin. 2025. “Functional diversification of epidithiodiketopiperazine methylation and oxidation towards pathogenic fungi.” *Mycology* 16: 1418-1431. https://doi.org/10.1080/21501203.2025.2496190

11.	Lehmann, Anika, Weishuang Zheng, Masahiro Ryo, Katharina Soutschek, Julien Roy, Rebecca Rongstock, Stefanie Maaß, et al. 2019. “Fungal traits important for soil aggregation.” *Frontiers in Microbiology* 10: 2904. https://doi.org/10.3389/fmicb.2019.02904

12.	Li, Juan, Jiujie Li, Lili Cao, Qinghua Chen, Ding Ding, Le Kang. 2024. “An iron-binding protein of entomopathogenic fungus suppresses the proliferation of host symbiotic bacteria.” *Microbiome* 12: 202. https://doi.org/10.1186/s40168-024-01928-4

13.	Jin, Xue, Huiting Jia, Lingyi Ran, Fengzhi Wu, Junjie Liu, Klaus Schlaeppi, Francisco Dini-Andreote, et al. 2024. “Fusaric acid mediates the assembly of disease-suppressive rhizosphere microbiota via induced shifts in plant root exudates.” *Nature Communications* 15: 5125. https://doi.org/10.1038/s41467-024-49218-9

14.	Fuchs, J. G., Y. Moënne-Loccoz, G. Défago. 1997. “Nonpathogenic *Fusarium oxysporum* strain Fo47 induces resistance to *Fusarium* wilt in tomato.” *Plant Disease* 81: 492-496. https://doi.org/10.1094/pdis.1997.81.5.492

15.	Hu, Jing, Effiong Kokoette, Caicai Xu, Shitao Huang, Tao Tang, Yiyi Zhang, Muyuan Liu, et al. 2023. “Natural algaecide sphingosines identified in hybrid straw decomposition driven by white-rot fungi.” *Advanced Science* 10: e2300569. https://doi.org/10.1002/advs.202300569

16.	Ma, Siqi, Qianru Chen, Yanfen Zheng, Tingting Ren, Rui He, Lirui Cheng, Ping Zou, et al. 2025. “A tale for two roles: Root-secreted methyl ferulate inhibits *P. nicotianae* and enriches the rhizosphere *Bacillus* against black shank disease in tobacco.” *Microbiome* 13: 33. https://doi.org/10.1186/s40168-024-02008-3

17.	Li, Shengjie, Damon Mosier, Xiaoli Dong, Angela Kouris, Guodong Ji, Marc Strous, Muhe Diao. 2023. “Frequency of change determines effectiveness of microbial response strategies.” *The* *ISME Journal* 17: 2047-2057. https://doi.org/10.1038/s41396-023-01515-9

18.	Masotti, Andrea, Tobias Preckel. 2006. “Analysis of small RNAs with the Agilent 2100 Bioanalyzer.” *Nature Methods* 3: 658-658. https://doi.org/10.1038/nmeth908

19.	Shin, Myung Geun, Jun Hyung Lee, Hyun Jung Choi, Seung Jung Kee, Soo Hyun Kim, Jong Hee Shin, Soon Pal Suh. 2018. “RNA sequencing based whole transcriptome analysis aetected precisely all fusion transcripts in Leukemias.” *Blood* 132: 1496. https://doi.org/10.1182/blood-2018-99-115785

20.	Waugh, Katherine A., Ross Minter, Jessica Baxter, Congwu Chi, Matthew D. Galbraith, Kathryn D. Tuttle, Neetha P. Eduthan, et al. 2023. “Triplication of the interferon receptor locus contributes to hallmarks of Down syndrome in a mouse model.” *Nature Genetics* 55: 1034-1047. https://doi.org/10.1038/s41588-023-01399-7

21.	Zhang, Jingying, YongXin Liu, Xiaoxuan Guo, Yuan Qin, Ruben Garrido-Oter, Paul Schulze-Lefert, Yang Bai. 2021. “High-throughput cultivation and identification of bacteria from the plant root microbiota.” *Nature Protocols* 16: 988-1012. https://doi.org/10.1038/s41596-020-00444-7

22.	Zhang, Ying, Kangyou Zhu, Xiujie Wang, Jiarong Yan, Haiyan Zhu, Nan Zhang, Yiting Wang, et al. 2025. “Manipulation of artificial light environment improves plant biomass and fruit nutritional quality in tomato.” *Journal of Advanced Research* 75: 79-93. https://doi.org/10.1016/j.jare.2024.11.030

23.	Liang, Lei, Cheng Kong, Jinming Li, Guang Liu, Jinwang Wei, Guan Wang, Qinying Wang, et al. 2024. “Distinct microbes, metabolites, and the host genome define the multi-omics profiles in right-sided and left-sided colon cancer.” *Microbiome* 12: 274. https://doi.org/10.1186/s40168-024-01987-7

24.	Ravikrishnan, Aarthi, Indrik Wijaya, Eileen Png, Kern Rei Chng, Eliza Xin Pei Ho, Amanda Hui Qi Ng, Ahmad Nazri Mohamed Naim, et al. 2024. “Gut metagenomes of Asian octogenarians reveal metabolic potential expansion and distinct microbial species associated with aging phenotypes.” *Nature Communications* 15: 7751. https://doi.org/10.1038/s41467-024-52097-9

**SUPPLEMENTARY FIGURES**

**Figure S1 Overview of the experimental design.** (A) Isolation and antifungal characterization of key bacterial strains, followed by whole-genome sequencing. (B) The construction of SynComs and in vitro screening of their inhibition against Fol. (C) Assessment of SynCom efficacy in soil solution system and pot experimengs under sterile and non-sterile conditions. (D) Screening of key differential metabolites and pot-based experiments to validate their anti-Fol activity. (E) Metatranscriptomic analysis and absolute qPCR quantification of key genes across treatments.

**Figure S2** **Effects of Fol infection on microbial community structure and inter-species interactions.** (A) Principal Coordinates Analysis (PCoA) plot based on Bray-Curtis distance. Green circles represent the Uninfected group, and orange triangles represent the Fol-infected group. (B) Spearman correlation heatmap of the top 50 most abundant genera (Fol infected). Red and blue colors indicate negative and positive correlations, respectively; color intensity and circle size represent the magnitude of the correlation coefficients (**p* < 0.05, ***p* < 0.01, ****p* < 0.001, n = 6).

**Figure S3** **Validation and evaluation of interbacterial antagonism.** V-shaped antagonistic assays between dual- and triple-strain combinations of *Bacillus/Paenibacillus* strains.

**Figure S4** **Evaluation of the inhibitory activity against Fol mycelial growth.** Quantification of mycelial growth inhibition across treatments. Values with different lowercase letters are significantly different, Tukey’s HSD test, *p* < 0.05, n = 5.

**Figure S5** **LMM spline-smoothed curves of estimated marginal means for AWCD (OD₅₉₀) over time under different treatments.** Curves represent estimated marginal means from a linear mixed-effects model (LMM) with restricted cubic spline fitting, showing overall temporal trends from 24 to 140 h.

**Figure S6** **Diversity index of carbon source utilization evaluated by the Biolog EcoPlate.** Diversity indices of carbon source utilization for each treatment (Shannon Diversity Index). Values with different lowercase letters are significantly different, Tukey’s HSD test, *p* < 0.05, n = 3.

**Figure S7** **Inhibitory effects of each treatment on Fol mycelium growth in the presence of microbial community from simulated soil suspension.** Verification of the inhibitory effect of synthetic flora on Fol under the background of simulated soil suspension. Values with different lowercase letters are significantly different, Tukey’s HSD test, *p* < 0.05, n = 5；Scale bar  = 1 cm..

**Figure S8** **Field results of the** **ABC SynCom inoculation on tomatoes and strawberries.** (A) Tomato plant growth status after application of ABC SynCom. (B) Tomato wilt disease incidence rate (Welch’s *t*-test, *p* < 0.01, n = 4). (C) Strawberry growth status after application of ABC SynCom. **p* < 0.05, ***p* < 0.01, and ****p* < 0.001.

**Figure S9 Determination of extracellular metabolites of bacteria in different treatment groups.** (A) Chemical composition of metabolites (top 10 classes shown), with color-coded classification and percentage representing proportional metabolite counts. (B) Metabolite clustering analysis and identification of differential metabolites. Clustered heatmap of the overall substances in the supernatants of each treatment. The relative abundance is indicated by different colors. The columns represent samples, and the rows represent metabolite names. The dendrogram on the left side of the figure is the clustering tree of differential metabolites.

**Figure S10** **Comparison of the major significantly different metabolites among different treatment groups.** LDA Effect Size analysis of metabolites in the supernatants of each treatment group to identify metabolites with significant abundance differences between groups, with an LDA score set at > 2.5, *p* < 0.05.

**Figure S11** **Five-fold cross-validation results based on the random forest model.** (A) The x-axis represents the number of variables included, and the y-axis indicates the cross-validation error. The line with data points shows the trend of error change across different model complexities. (B) Based on the cross-validation curve, four important variables were selected for retention to achieve satisfactory regression performance.Variable importance of metabolites in predicting the inhibition rate of Fol based on random forest regression. The y-axis represents the increase in mean squared error (%IncMSE), indicating the contribution of each metabolite to the model prediction. Asterisks denote significant variables based on 1,000 permutations (**p* < 0.05, ***p* < 0.01, ****p* < 0.001).

**Figure S12** **Antifungal activity of selected metabolites against Fol.** Evaluation of inhibitory effects of various metabolites on Fol mycelial growth. Values with different lowercase letters are significantly different, Tukey’s HSD test, *p* < 0.05, n = 5.

**Figure S13** **Cost comparison between vitamin K2 and vitamin K3.**

**Figure S14 Comparison of tomato plant biomass among different treatments.** (A) Aboveground biomass of tomato plants under vitamin K3 treatment. (B) Belowground biomass of tomato plants under vitamin K3 treatment. Data are presented as mean ± s.e.m. Different letters indicate significant differences among treatments. Values with different lowercase letters are significantly different, one-way ANOVA with Tukey’s HSD test, *p* < 0.05, n = 3.

**Figure S15 Functional gene annotation of *Bacillus amyloliquefaciens* GXJ-12.** (A) GO annotation pathways classification of *Bacillus amyloliquefaciens* GXJ-12. (B) KEGG annotation pathways classification of *Bacillus amyloliquefaciens* GXJ-12.

**Figure S16** **Functional gene annotation of *Paenibacillus polymyxa* ZLZ-12.** (A) GO annotation pathways classificationof *Paenibacillus polymyxa* ZLZ-12. (B) KEGG annotation pathways classificationof *Paenibacillus polymyxa* ZLZ-12.

**Figure S17 Functional gene annotation of *Bacillus megaterium* YQF-12.** (A) GO annotation pathways classification of *Bacillus megaterium* YQF-12. (B) KEGG annotation pathways classification of *Bacillus megaterium* YQF-12.

**Figure S18** **Standard curves for absolute quantification of genes.** Standard curves for absolute quantification of genes in the *Men/Mqn* cluster.

**Figure S19** **Melting curves for absolute quantification of genes.** Melting curves for absolute quantification of genes in the *Men/Mqn* cluster.
